# Exploring the Influence of Origin, Harvest Time, and Cultivation Method on Antioxidant Capacity and Bioactive Compounds of Matcha Teas

**DOI:** 10.3390/foods13081270

**Published:** 2024-04-21

**Authors:** Karolina Jakubczyk, Kinga Szymczykowska, Joanna Kika, Katarzyna Janda-Milczarek, Joanna Palma, Klaudia Melkis, Rami Alshekh, Dominika Maciejewska-Markiewicz

**Affiliations:** 1 Department of Human Nutrition and Metabolomics, Pomeranian Medical University in Szczecin, 24 Broniewskiego Street, 71-460 Szczecin, Poland; kinga.szymczykowska@pum.edu.pl (K.S.); joanna.kochman@pum.edu.pl (J.K.); katarzyna.janda.milczarek@pum.edu.pl (K.J.-M.); 58147@student.pum.edu.pl (K.M.); info@dr-rami.pl (R.A.); dominika.maciejewska@pum.edu.pl (D.M.-M.); 2 Department of Biochemical Science, Pomeranian Medical University in Szczecin, 71-460 Szczecin, Poland; joanna.palma@pum.edu.pl

**Keywords:** Matcha, powdered green tea, antioxidants, polyphenols, vitamin C, caffeine

## Abstract

Matcha, or powdered green tea, has been gaining popularity and is no longer consumed only in the form of infusions, finding new uses in gastronomy and the food industry. The range of teas available on the food market has expanded considerably; hence, the aim of this study was to determine, for the first time, the antioxidant capacity and contents of antioxidant compounds in various Matcha teas available on the Polish market, taking into account the country of origin, time of harvest, and conventional vs. organic cultivation. Eleven green-tea powders were used in the analyses performed using spectrophotometric methods (Trolox equivalent antioxidant capacity, Ferric-Ion-Reducing Antioxidant Power, Total Polyphenol Content, Total Flavonoid Content, Vitamin C Content) and HPLC methods (polyphenolic acids, flavonoids, and caffeine). Antioxidant capacity ranged from 7.26 to 9.54 mM Trolox equivalent/L while reducing power ranged from 1845.45 to 2266.12 Fe(II)/L. Total phenolic content amounted to 820.73–1017.83 mg gallic acid equivalent/L, and total flavonoid content was 864.71–1034.40 mg rutin equivalent /L. A high vitamin C content was found, ranging from 38.92 to 70.15 mg/100 mL. Additionally, a high content of caffeine that ranged between 823.23 and 7313.22 mg/L was noted. Moreover, a high content of polyphenolic compounds, including epicatechin gallate, myricetin, gallic acid, and 4—hydroxybenzoic acid, was found. The phytochemical composition and antioxidant properties depended on the harvest time, type of cultivation, and country of origin. Therefore, Matcha tea infusions have been shown to be a valuable source of antioxidants that can be used in the daily diet.

## 1. Introduction

Tea (*Camellia sinensis* L.) is one of the most widely consumed beverages in the world, coming in many types (green, black) and forms (bags, leaves or powdered) [1]. Originally from Asia (regions of China and India), it is now produced in many other countries, too [2]. One of its types is green tea, which is available on the market in the form of granules, leaves of different sizes, and powder [3]. Green tea is characterised by a particularly high content of bioactive compounds and multiple health-promoting properties [1,4]. The most powerful antioxidants in green teas are believed to be representatives of the phenolic family, such as phenolic acids and flavonoids, including flavanols, proanthocyanidins, isoflavones, and flavonols [5], along with carotenoids, tocopherols, vitamin C, and minerals (Cr, Mn, Se, and Zn). The antioxidant mechanism of these compounds involves enhancing the activity of antioxidant enzymes, e.g., glutathione transferase (GT) and superoxide dismutase (SOD); inhibiting lipid peroxidation; scavenging free radicals; and suppressing oxidation by chelating metal ions [6,7,8]. Importantly, green tea shows stronger antioxidant activity against superoxide radicals than vegetables with high antioxidant potentials (Brussels sprouts, garlic, kale, and spinach) [7,9]. Out of all the polyphenols, many of the health benefits of tea can be attributed to epigallocatechin gallate [10], which exhibits strong antioxidant properties [11,12]. Thanks to its functional bioactive substances, green tea is useful in prevention and delaying the onset of cardiovascular diseases, neurodegenerative diseases, and type 2 diabetes, as well as for weight control [13,14]. The anti-inflammatory, anti-viral, and detoxifying effects of green tea have also been demonstrated [15]. In addition, green tea is capable of stimulating the body’s immune function and inhibiting platelet aggregation [10].

Matcha, or powdered green tea, is becoming increasingly popular [16]. Unlike other types of tea, Matcha is consumed in powdered form, containing all parts of the leaf [17]. It is cultivated using a unique traditional method, shaded under bamboo mats. The tea plants remain shaded for approximately 21 days before collection [18]. This cultivation method means that the young leaves are protected from the sun’s rays, resulting in increased contents of amino acids (mainly theanine) and caffeine and lower contents of catechins compared to other green teas [18]. The proportions of these ingredients impart a distinctive ‘umami’ flavour to Matcha [13]. Matcha is a relative newcomer to the market, despite its long tradition, and there are few detailed reports on its composition and health-promoting properties. However, recent research studies highlight the antioxidant properties of Matcha infusions [7,13,14,16,17,19], positive effects on cognitive function, and anti-inflammatory properties [16,18,20,21,22,23]. Matcha tea is distinguished not only by its high contents of bioactive compounds but also by its better bioavailability compared to regular green tea. Rusak et al. investigated both the total contents of polyphenols, flavonoids, and flavanols in dried tea as well as the bioavailability of Matcha compared to sencha tea. Total contents of phenols, flavonoids, and flavanols and antioxidant and antidiabetic activity (α-glucosidase inhibition) were higher in Matcha tea. Interestingly, upon gastric digestion, there was an increase in the occurrence of bioactive components, and antioxidant activity was significantly (*p* ≤ 0.05) higher—by 2.4 and 2.0 times with ABTS and FRAP, respectively—whereas α-glucosidase inhibition was 1.7 times significantly (*p* ≤ 0.05) higher in Matcha tea. For the first time, it was shown that when Matcha powder is digested together with its water extract, Matcha polyphenols become more bioavailable and have higher antioxidant and antidiabetic activity compared to Sencha [24]. Matcha appears to be one of the most potent antioxidants in beverages, especially teas, and, importantly, it is most often consumed in liquid form, which makes it easy to incorporate into the daily diet. 

However, it needs to be emphasised that antioxidant properties depend not only on the species of plant. Geographical conditions and cultivation and processing methods can significantly affect the concentration of antioxidants in the finished food product. Moreover, in order to derive the most benefits from Matcha, it is important to brew it using water at the right temperature [5,13]. Apart from the brewing temperature, the health properties of tea are also influenced by the solvent in which the infusion or extract is prepared, as other studies have proven [17,25]. Moreover, with the use of solvents other than water and different extraction techniques for the raw material, a unique composition of antioxidant compounds can be obtained [26,27]. The quality of the raw material is also affected by temperature and storage time. It is important to store Matcha for as short a time duration as possible at a temperature of 0–5 °C. Then, it retains its properties, and there is no loss of bioactive compounds [28,29]. The present study aimed to establish the antioxidant capacity and levels of compounds with antioxidant effects in different Matcha teas available on the Polish market, taking into account the country of origin, time of harvest, and method of cultivation (conventional vs. organic). Testing the antioxidant potential of Matcha tea infusions based on country of origin, time of harvest, and cultivation method is a novel approach that can be helpful to both consumers and researchers. Knowing the influence of the following factors on the chemical composition and antioxidant properties will enable consumers to make informed choices regarding what constitutes good quality Matcha. The study results will optimise quality parameters, which will increase the health-promoting properties of Matcha and facilitate the creation of functional foods with this ingredient.

## 2. Materials and Methods

The study material consisted of 11 Matcha teas available in the Polish food market, purchased from various suppliers (Table S1, Supplementary Materials). The teas came from three countries: Japan, China, and South Korea. Table S1 (available in Supplementary Materials) shows the characteristics of the study material in terms of BIO certification (conventional vs. organic cultivation), harvest time, and country of origin (Table S1, Supplementary Materials). Green tea harvest (chatsumi) begins in late April. The first harvest, or spring harvest, lasts until the end of May. After the leaves are plucked, new buds start to grow in their place and are ready for harvesting in late June/early July (second harvest). The third harvest takes place in August. Tea from each subsequent harvest differs in terms of biochemical composition and organoleptic qualities. The ‘Summer’ tea group includes teas harvested from May to late June/early July and from late June/early July to August while the ‘Spring’ tea group includes teas harvested only in May.

### 2.1. Preparation of Infusion 

An aliquot of 1.75 g of Matcha was placed in a conical flask with 100.0 mL of distilled water heated to a 90 °C temperature (used to prepare plant infusions most commonly) [13]. Infusions in the flasks were sealed and shaken at a speed of 180 rpm for 10 min. (laboratory shaker, Brunswick model EXCELLA E24). After this time, the Matcha residues were separated through filtration from the infusion. The pore size was 0.22 µm. Analyses were carried out immediately after the infusions were cooled to room temperature [7,13]. Infusions of all Matcha samples were performed in repetitions (three) and each assay was repeated three times. 

### 2.2. Antioxidant Activity, Measured via the Trolox Equivalent Antioxidant Capacity (TEAC Method)

The spectrophotometric method, using synthetic radical DPPH (2.2-diphenyl-1-picrylhydrazyl, Sigma, Poznań, Poland) according to Brand-Williams et al. and Pekkarinen et al., was used to measure the antioxidant activity of samples [30,31]. Quantities of 0.1 mL of the test sample and 1 mL of 0.3 mM solution of DPPH in 96% ethanol and 96% ethanol in *v*/*v* ratio 29:10:1 were transferred into the vial. After mixing, the prepared solution was incubated in a dark place for 30 min. The so-called A0 solution was prepared by mixing 96% ethanol and 0.3 mM solution of DPPH in *v*/*v* ratio 3:1 during the time of incubation. As a reference solution, 96% ethanol was used. The vial contents were thoroughly mixed and poured into cuvettes before the measurement [32].

Immediately, the spectral absorbance was measured at 518 nm (8453UV, AGILENT TECHNOLOGIES, Santa Clara, CA, USA). To run the calibration curve, the range of Trolox concentrations used was as follows: 1, 0.6, 0.2, 0.12 uM Trolox. All assays were performed in triplicate. The results are shown in Trolox equivalent as the reference standard.

### 2.3. Determination of the Ferric-Ion-Reducing Antioxidant Power (FRAP Method) 

The FRAP method is based on the ability of the test sample to reduce Fe^3+^ ions to Fe^2+^ ions. It is used to determine the total reduction potential, which also means the antioxidant properties of tested ingredient [33]. A FRAP unit corresponds to the ability to reduce 1 micromole Fe^3+^ to Fe^2+^ according to Benzie and Strain [34,35]. A quantity of 3 mL of the FRAP reagent consists of 10 mM tripyridyltriazine (TPTZ) solution, 300 mM acetate buffer (pH = 3.6), and 20 mM FeCl_3_. It was added with the test sample and distilled water into the vial. After mixing, the prepared solution was placed for 5 min at 37 °C. The absorbance was measured at 593 nm (8453UV, AGILENT TECHNOLOGIES, Santa Clara, CA, USA). All assays were performed in triplicate. The ferric-ion-reducing antioxidant power was determined from the calibration curve using Fe(II)/L as the reference standard (0–5000 µM Fe(II)/L) [33]. 

### 2.4. The Determination of the Total Polyphenol Content (TPC)

According to Singleton and Rossi’s method, the Folin–Ciocalteu reagent was used to determine polyphenol content [36]; 5.0 mL of a Folin–Ciocalteu solution (10%) and 1.0 mL of test sample were added into the vial. The vial contents were thoroughly mixed, and 4.0 mL of 7.5% Na_2_CO_3_ solution was added after 5 min and incubated for 60 min at room temperature. To prepare reference solution, the same reagents were used, but instead of tested sample, distilled water was added. The absorbance was measured at 765 nm (8453UV, AGILENT TECHNOLOGIES, Santa Clara, CA, USA). All assays were performed in triplicate. The content of polyphenols was determined from the calibration curve using gallic acid (GAE) as the reference standard (0–200 mg/L of gallic acid) [33]. 

### 2.5. The Determination of the Total Flavonoid Content (TFC)

Pękal and Pyrzynska and Hu methods were used to determine the total flavonoid content [37,38]. Firstly, 0.6 mL of an NaNO_2_ solution (5%) and 2.0 mL of test sample were added into the vial. The vial contents were thoroughly mixed and incubated for 6 min, then 0.5 mL of AlCl_3_ solution (10%) was added. Incubation was repeated under the same conditions. A 4.3% NaOH solution was added, and the 10 mL flask was filled to the line with distilled water. Using rutin equivalent as a reference standard (0–120 mg/L rutin equivalent), the flavonoid content was determined from the calibration curve. The absorbance was measured at 510 nm (8453UV, AGILENT TECHNOLOGIES, Santa Clara, CA, USA). All assays were performed in triplicate [33]. 

### 2.6. The Determination of the Vitamin C Content

The content of vitamin C was measured using the spectrophotometric method with the use of a dye, 2.6 dichlorophenolindophenol (2,6-DCPIP; DCPIP). The test sample and oxalic acid were added to the dark glass flask. Then, the acetate buffer solution (pH = 4) and dye (2,6-DCPIP) were added. After mixing, 10 mL of xylene was added to the sample. The flask was closed again and shaken for 10 s. The sample was then set aside until the organic xylene layer separated in the sample. Different concentrations of dye 2.6 dichlorophenolindophenol were used in the plotting of the standard calibration curve. The absorbance was measured at 510 nm (8453UV, AGILENT TECHNOLOGIES, Santa Clara, CA, USA). All assays were carried out in triplicate. 

### 2.7. The Determination of the Phenolic Acid and Caffeine Contents

Liquid chromatography (Agilent Technologies 1260 HPLC System, USA) was used in order to determinate polyphenol compounds. Hypersil Gold (150 × 4.6) column was used with the temperature maintained at 25 °C. Phenolic compounds were detected through UV absorption at λ = 278 nm. Retention times and comparison with standards under the same conditions were used to identify each compound. The mobile phase consisted of 1% aqueous acetic acid solution (A) and 100% MeOH (B). The following gradient was used to elute the samples: 90% A and 10% B from 0 to 6 min, 84% A and 16% B from 7 to 25 min, 72% A and 28% B from 26 to 37 min, 65% A and 35% B from 38 to 47 min, 50% A and 50% B from 48 to 64 min, and 90% A and 10% B from 65 to 70 min. This gradient was used to restore the initial conditions before injection of a new sample. The flow rate was 0.8 mL/min, and the injection volume was 30 µL. According to the determination of the calibration curve, the LOD was set at 0.1 ppm level and the LOQ was set at 0.75 ppm level.

### 2.8. Statistical Analysis

All determinations were carried out in three replicates. StatSoft Statistica 13.0 (StatSoft Polska Sp. z o.o., Kraszewskiego Street 36, 30-110, Kraków, Poland) and Microsoft Excel 2017 were used to perform statistical analysis. Distributions of values for individual parameters were analysed using the Shapiro–Wilk test. The Kruskal–Wallis test was used to evaluate the differences between the studied parameters as the distribution of continuous variables deviated from normal. To determine the correlations between the parameters studied, Spearman’s correlation test was used. Sparse PLS exploratory approach (CIT: https://bmcbioinformatics.biomedcentral.com/articles/10.1186/1471-2105-12-253, accessed on 11 January 2024) was used to perform variable selection in a multiclass (origin) classification framework. Results were expressed as mean values and standard deviations; however, median values and quartile ranges were used for statistical analyses. Differences were considered significant at *p* ≤ 0.05.

## 3. Results

The tested infusions had high antioxidant potentials in the range of 7.11–10.43 mM/L Trolox equivalent and reducing potentials in the range of 13,452.44–26,258.33 mM Fe(II)/L. Differences between samples are highlighted in Table 1. Matcha’s high antioxidant potential is related to the presence of numerous bioactive compounds found in the brew. The green tea powder infusions had a high content of polyphenols (671.29–1083.13 mg/L gallic acid equivalent), mainly flavonoids (352.36–1359.70 mg/L rutin equivalent). High levels of vitamin C were also found in the infusions, ranging from 37.38 to 76.17 mg/100 mL (Table 1). The Recommended Dietary Allowance (RDA) intake for an adult is between 75 and 90 mg per day. Thus, just 100 mL of infusion can cover the daily requirement for this vitamin by up to 84%.

In addition, we examined the biochemical composition and antioxidant properties depending on the cultivation method. Our study showed differences associated with the cultivation method—organic vs. conventional. Statistically significant differences were noted in the contents of polyphenols and vitamin C and antioxidant capacity, measured using the TEAC method. Antioxidant activity in the tested infusions, expressed as ferric-reducing antioxidant power, fell in the range between 2112.96 and 1845.45 mM Fe(II)/L. Higher levels were observed in organic crops. TEAC scores ranged from 8.94 to 8.25 Trolox (mM/L). In this case, higher values were found in conventional crops. Polyphenol concentrations ranged from 963.95 to 820.73 mg/L. Higher values were observed in organic crops (Table 2). The infusions also had a high content of flavonoids, which are a class of polyphenols. Higher concentrations of these compounds were found in conventional crops, but the differences between them and organic ones were not statistically significant. Furthermore, Matcha in infusion form is an excellent source of vitamin C. The content of this vitamin in Matcha infusions ranged from 41.66 to 61.37 mg/100 mL (Table 2). According to nutritional standards, the recommended daily intake of vitamin C is 90 mg for adult men and 75 mg for adult women, so 100 mL of this infusion will cover as much as 82% of the requirement in the case of women and 68% for men. Our results show that organically grown Matcha has high contents of antioxidants, polyphenols, and vitamin C.

The highest polyphenol content was found in Matcha of unknown harvest time, and the lowest in that from summer harvests. With respect to flavonoids, the lowest concentrations were found in samples of unknown origin and the highest in the infusion prepared from tea leaves harvested in spring (Table 3). There were no statistically significant differences between the spring harvest and the summer harvest. We did, however, observe statistically significant differences between the summer and unknown harvests. In terms of antioxidant capacity and reducing power, higher values were obtained for infusions of unknown origin. Interestingly, the highest concentration of vitamin C was recorded for the product of the spring harvest (70.15 mg/100 mL). Statistically significant differences are highlighted in the table (Table 3). For these parameters, no differences were noted between the spring and summer harvests.

**Figure 1:**
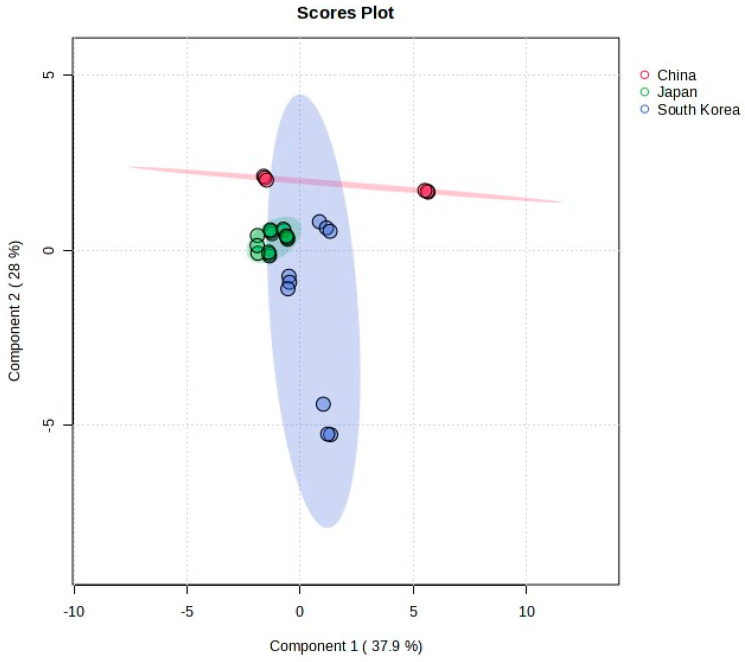
Geographic origin of Matcha.

The highest concentration of polyphenols was noted in Matcha from China and the lowest in Matcha from Japan (887.49 mg/L). In the case of flavonoids, which belong to the polyphenol group, the highest concentration was recorded in tea from Japan (975.04 mg/L) and the lowest in tea from China (912.08 mg/L), but these results were not statistically significant. High antioxidant capacity (9.54–7.65 mM/L) and reducing power (2266.12–1882.49 Fe(II)mM/L) were also noted. In both cases, the highest value was observed in infusions prepared with tea leaves from China and the lowest in those from Japan. Interestingly, the highest concentration of vitamin C was found in the product from Japan: 66.37 mg/100 mL. It can therefore be concluded that Matcha brewed with powder from China exhibits the most powerful antioxidant activity, due to the presence of polyphenolic compounds, while the product from Japan is rich in vitamin C and flavonoids (Table 4). Statistically significant differences are presented in Table 4. The sparse PLS method visualised the most common features existing in Japan samples (Figure 1).

There was a fairly strong positive correlation between FRAP and polyphenol content, as well as a moderate correlation between TEAC and polyphenol content. This finding confirms that a higher polyphenol content in the infusions is associated with more powerful antioxidant activity. A moderate positive relationship was also noted between the antioxidant parameters TEAC and FRAP. Thus, both methods of measuring antioxidant activity in plant material produced consistent results. In addition, strong positive correlations were noted between chlorogenic acid and 4-hydroxybenzoic acid and between p-coumaric acid and ferulic acid (and also gallic acid). Compounds that correlated strongly with resveratrol content were 4-hydroxybenzoic acid and chlorogenic acid, and those that correlated strongly with quercetin were ferulic acid, gallic acid, and p-coumaric acid. Sinapic acid correlated strongly with ellagic acid and resveratrol while rutin correlated with ferulic acid, gallic acid, and p-coumaric acid and also with quercetin. All correlations are shown in Table S2, available in the Supplementary Materials.

**Figure 2:**
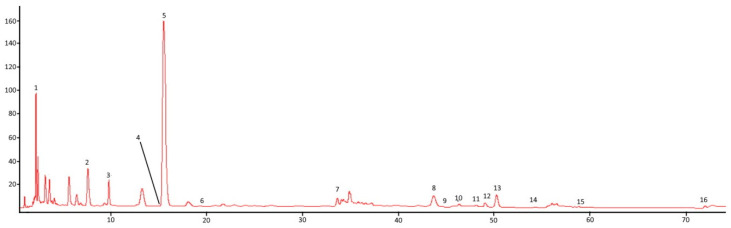
Chromatograph of polyphenolic acids, flavonoids, and caffeine in Matcha: ^1^ gallic acid, ^2^ 4-hydroxybenzoic acid, ^3^ chlorogenic acid, ^4^ caffeic acid, ^5^ caffeine, ^6^ p-coumaric acid, ^7^ ferulic acid, ^8^ epicatechin gallate, ^9^ sinapic acid, ^10^ ellagic acid, ^11^ rutin, ^12^ resveratrol, ^13^ myricetin, ^14^ quercetin, ^15^ kaempferol, and ^16^ apigenin.

The present study also examined the quantitative and qualitative composition of polyphenolic compounds in 11 samples of Matcha. The presence of 16 compounds, including the following groups, was noted in the analysed material: phenolic acids (gallic acid, 4-hydroxybenzoic acid, p-coumaric acid, ferulic acid, sinapic acid, ellagic acid), flavonoids (epicatechin gallate, rutin, resveratrol, myrcetin, quercetin, kaempferol, apigenin), and caffeine. Of the phenolic acids, the highest content of gallic acid was shown (median 153.01 mg/L), and of the flavonoids, that of epicatechin gallate (median 165.79 mg/L) (Figure 2). However, high levels of 4-hydroxybenzoic acid (median 50.02 mg/L) and myricetin (median 105.23 mg/L) were also detected (Table S3, Supplementary Materials). The polyphenolic acids identified in Matcha, especially chlorogenic acid or gallic acid, are characterised by high antioxidant potential, which may determine the properties of this brew. 

In addition, the study analysed the caffeine content of Matcha. Its content ranged from 826.23 mg/L to 7313.22 mg/L, and the median was 1853.20 mg/L (Table S3, Supplementary Materials). Consequently, a cup of Matcha (100 mL) will provide about 8–70 mg of caffeine. 

In the present study, the contents of the polyphenolic compounds and caffeine, tested in terms of Matcha’s place of origin, were compared. The results showed that the country of origin influences the content of compounds such as caffeic acid, ellagic acid, sinapic acid, kaempferol, resveratrol, rutin, and caffeine. The highest concentrations of most compounds, including caffeic acid, ellagic acid, sinapic acid, resveratrol, and caffeine, were recorded for Matcha from South Korea. Concentrations of kaempferol and rutin were highest in samples from China. No statistically significant differences were observed for the other compounds (Table 5).

Matchas, divided as per cultivation method, showed statistically significant differences for ellagic acid, sinapic acid, myricetin, apigenin, resveratrol, and caffeine. Organic teas have been shown to be a better source of these compounds compared to conventionally grown teas (Table 6). 

Matchas divided as per terms of time of harvest showed statistically significant differences for caffeic acid, kaempferol, quercetin, rutin, and caffeine. Matchas from different harvests differed in their polyphenol and caffeine contents. For caffeine, the highest content was recorded for the summer harvest (2336.11 mg/L), with no statistically significant differences between spring (1802.66 mg/L) and unknown (1802.62 mg/L) harvests. Caffeine content was the highest among the contents of the other compounds tested. For flavonoids, including rutin, quercetin, and kaempferol, significantly higher concentrations were recorded for samples harvested in summer while the lowest concentrations were recorded in spring, with rutin present only in one sample from the spring harvest. For caffeic acid, higher concentrations were recorded for samples of unknown origin (Table 7).

## 4. Discussion

Out of all the tea forms, Matcha appears to be the most potent antioxidant. Both its antioxidant potential and polyphenol content are several times higher than in other teas. Recent scientific studies confirm the high contents of polyphenols and other antioxidant compounds in green teas including Matcha [4,7,39]. Therefore, the aim of this study was to determine, for the first time, the antioxidant capacity and the contents of antioxidant compounds in different Matcha teas available on the Polish market, taking into account country of origin, harvest time, and conventional and organic cultivation. In this study, the infusions had antioxidant potentials in the range of 7.11–10.43 mM/L Trolox equivalent and reducing potentials in the range of 13,452.44–26,258.33 mM Fe(II)/L. Matcha infusions had a high content of polyphenols (671.29–1083.13 mg/L gallic acid equivalent), mainly flavonoids (352.36–1359.70 mg/L rutin equivalent). Organically grown teas were found to have significantly higher contents of vitamin C and polyphenols and more potent reducing power (FRAP). These differences may be attributable to the different growing conditions. Organically grown teas do not contain residues of chemical pesticides and are not genetically modified [40]. In addition, products of organic cultivation are selected and of high quality. The study included a small number of samples from conventional farming, due to the popularity of organic products available in the food market, indicating high awareness among producers and the intention to provide the highest-quality products possible.

The highest concentration of polyphenols was noted in Matcha from China (1017.83 mg/L) and the lowest in Matcha from Japan (887.49 mg/L). The difference was statistically significant. This regularity was confirmed in a study by Bobková et al. wherein the researchers analysed green teas from China, Korea, and Japan, with similar observations [41]. Maslov et al., in their examination of the polyphenol content in green teas from China, obtained lower results, which may have been the result of both differences in methodology as well as the selection of leaves already at the stage of cultivation and processing [42]. In the case of flavonoids, which belong to the polyphenol group, an inverse relationship was noted. The highest concentration was recorded in tea from Japan (975.04 mg/L) and the lowest in tea from China (912.08 mg/L), but these results were not statistically significant. High antioxidant capacity (9.54–7.65 mM/L) and reducing power (2266.12–1882.49 Fe(II)mM/L) were also noted. In both cases, the highest value was observed in infusions prepared with tea leaves from China and the lowest in those from Japan. Interestingly, the highest concentration of vitamin C was found in the product from Japan: 66.37 mg/100 mL [42]. 

Our results also confirm that harvest time had a significant effect on the phytochemical composition and antioxidant capacity of the infusions. Matcha available on the food market comes from different harvests, but the relevant information is not always provided on the label. 

Matchas of unknown harvest time are the ones with the highest polyphenol content, and the lowest are the ones from summer harvests. With respect to flavonoids, the lowest concentrations were found in samples of unknown origin and the highest in the infusion prepared from tea leaves harvested in spring. There were no statistically significant differences between the spring harvest and the summer harvest. We did, however, observe statistically significant differences between the summer and unknown harvests. In terms of antioxidant capacity and reducing power, higher values were obtained for infusions of unknown origin. Interestingly, the highest concentration of vitamin C was recorded for the product of the spring harvest (70.15 mg/100 mL). For these parameters, no differences were noted between the spring and summer harvests. These results confirm that Matcha of unknown origin is not an inferior product in terms of nutritional quality but, nevertheless, information regarding the harvest time on product labels would be valuable. Interestingly, He, G. et al. examined the polyphenolic content in green teas harvested at different times of the year. Gallic acid content was significantly higher in teas harvested in spring than in those harvested at other times of the year [43]. These results confirm that this research should be continued and that teas from all countries are valuable sources of antioxidants.

In our earlier study, we demonstrated both a high polyphenol content as well as strong antioxidant properties in Matcha infusions prepared in the same way [13]. The aim of that study was to determine the antioxidant potential and contents of antioxidant substances—vitamin C and total polyphenols including flavonoids—in infusions of Traditional Matcha (from the first and second harvests) and Daily Matcha (from the second and third harvests) made at different temperatures. Very high antioxidant activity was found. According to the FRAP method, which was also applied in this study, the reducing power amounted to 612.95 mM Fe(II)/L. In a subsequent test, high levels of antioxidant substances were observed in this infusion (flavonoids: 1968.8 mg/L; polyphenols: 1765.1 mg/L) [13]. High polyphenol content has also been confirmed by other researchers [17,44]. These results are consistent with the present study.

Based on the results of other authors, the antioxidant properties of Matcha can be attributed mainly to a group of flavonoids known as catechins, the main representatives being epicatechin (EC), epigallocatechin (EGC), and epigallocatechin gallate (EGCG) [45]. Catechins have been proven to be the most efficient contents of green teas in reducing radicals [46]. The present study confirmed that Matcha infusions are an excellent source of epicatechin gallate: its content ranged from 2.68 mg/L to 234.03 mg/L. Despite large differences for the individual samples, no influence of factors such as country of origin, harvest, or type of cultivation was demonstrated. The high content of polyphenols, including catechins, was confirmed by Koláčková et al. Catechins were confirmed to have the best-released of the polyphenolic compounds included in the study [45]. In the study by Komes et al., the epicatechin gallate content in the Matcha tea samples was as high as 102.67 mg/L of water extract [5]. Meanwhile, in the study by Unno et al., the authors examined fresh Matcha powder, and the content of epicatechin gallate was 27.96 mg/g [18]. Meyer et al. confirmed Matcha’s catechin content, including its high levels of epicatechin gallate, which was found to be as high as 11.89 mg/g [44].

Apart from the polyphenols, it has been confirmed that Matcha has a high content of vitamin C, which, in addition to its antioxidant properties, effectively reduces triglycerides in the body and inhibits the activity of glycerol-3-phosphate dehydrogenase [47]. By way of comparison, Jakubczyk et al. also examined the vitamin C contents of Matcha teas, which ranged from 32.12 to 44.8 mg/L. Koláčková et al., too, examined the vitamin C contents of various Matcha teas, reporting vitamin C concentrations ranging from 1.63 to 3.98 mg/g [17]. In turn, Park et al. reported vitamin C content in green tea in the range of 1.35–1.53 mg/g [48]. Compared to other teas, Matcha is also distinguished by its high content of vitamin C, which is several times higher than in other varieties [13,49,50]. The different results may be due to the fact that a less accurate Tillmans’ method of titration was used in the study by Jakubczyk et al. Other factors to be taken into account include the quality of the input product and its storage conditions [13]. It is important, however, that Matcha is a significant source of vitamin C in the diet, and a cup of the beverage represents as much as 94% of the vitamin C requirement for an adult woman and 79% of that for a man. It should be emphasised that the phytochemical composition of Matcha is not fully understood, and, importantly, the impact of additional parameters, such as country of origin, cultivation method, and harvest time, has been presented here for the first time.

This study was the first to examine the contents of so many flavonoids and phenolic acids. For the first time, qualification in terms of cultivation method, country of origin, and time of harvest was also included. The medians of the contents of other flavonoids in the present study were as follows: myricetin—105.23 mg/L, resveratrol—16.71 mg/L, rutin—14.12 mg/L, apigenin—8.72 mg/L, and kaempferol—1.52 mg/L.

Rutin and quercetin are powerful antioxidants, meaning that these can neutralise free radicals in plant cells. The antioxidant properties of these compounds help protect plants from oxidative stress, which can be caused by various factors such as UV radiation, pollution, or temperature changes. Thus, flavonoids can act as a natural UV filter for plants, especially during sunny periods, protecting a plant from the harmful effects of UV radiation. In the present study, Matcha green tea harvested in summer was found to contain significantly higher levels of both caffeine as well as flavonoids including rutin, quercetin, and kaempferol, which may have been related to the higher production of phytochemicals during this period in response to very high sunlight. It is also worth noting that the chemical composition of tea can be complex and dependent on a number of factors including the growing region and soil and climatic conditions that may be related to the country of origin of the raw material. In the study, we confirmed that more caffeine and polyphenolic acids including caffeic acid, ellagic acid, sinapic acid, and resveratrol were recorded for Matcha sourced from South Korea. In contrast, samples from China contained significantly more kaempferol and rutin. However, more research is needed to confirm which factors may determine the chemical composition of Matcha. It also seems important for manufacturers to include information such as region and altitude on Matcha labels. In addition, the grinding and storage process of the dried Matcha itself can also affect the chemical composition. Therefore, differences in the contents of phytochemicals including rutin between Matcha teas harvested at different times of the year may be due to a combination of these factors.

Matcha is known for its strong health-promoting properties, particularly antioxidant properties, which are related to the presence of flavonoids in Matcha. However, these compounds exhibit many health-promoting properties. Myricetin, among others, shows anti-ageing and anti-cancer effects, prevents platelet aggregation, and has anti-hypertensive, immunomodulatory, anti-allergic, and analgesic effects [51]. Rutin is best known for its antioxidant action, focusing on reducing free radicals in the body. It shows blood-vessel-sealing, anti-edema, anti-inflammatory, and anti-infective effects. Resveratrol shows health benefits for chronic diseases including cardiovascular disease (reduces CVD risk factors), liver disease (e.g., MAFLD), obesity, diabetes, and neurodegenerative diseases (Alzheimer’s disease and Parkinson’s disease) [52]. Apigenin and its derivatives have been attributed with several beneficial properties including antioxidant, anti-inflammatory, and anti-cancer effects. Recent scientific studies indicate that apigenin protects against cancer, cardiovascular disease, arthritis, and diabetes [53].

Other authors have also shown that Matcha is an excellent source of flavonoids. Kaempferol content ranged from 4.2 to 20.4 μg/g of weight in 80% MeOH extracts and 1.7–16.2 μg/g of weight in water extracts. Quercetin content ranged from 8.4 to 17.2 μg/g of weight in 80% MeOH extracts and 10.5–84.9 μg/g of weight in water extracts, and the rutin content was 570–2870 μg/g of weight in 80% MeOH extracts and 361–1590 μg/g of weight in water extracts. The highest contents of flavonoids (99–139 mg RE/g) and phenolics (169–273 mg GAE/g) were determined in methanol fractions [17]. 

The medians of the contents of phenolic acids in our study were as follows: gallic acid—153.01 mg/L, 4-hydroxybenzoic acid—50.02 mg/L, chlorogenic acid—32.54 mg/L, sinapic acid 30.80 mg/L, ferulic acid—13.83 mg/L, caffeic acid—3.51 mg/L, ellagic acid—9.17 mg/L, and p-coumaric acid—0.20 mg/L. Hydroxybenzoic acid’s derivatives include gallic acid and 4-hydroxybenzoic acid. The health-promoting effects of gallic acid are mainly attributed to its antioxidant and anti-inflammatory properties [54]. Hydroxycinnamic acid derivatives include caffeic acid, ferulic acid, synapic acid, and coumaric acid. Chlorogenic acid is also a derivative of cinnamic acid and is formed by combining the carboxyl group of caffeic acid with the phenolic group of quinic acid. It exhibits potent antioxidant, anti-inflammatory, antibacterial, and antiviral activities. It is postulated that it may also play a key role in regulating glucose and lipid metabolism, thereby reducing the risk of hypertension, atherosclerosis, heart failure, myocardial infarction, and other factors associated with cardiovascular risk, i.e., obesity or type 2 diabetes [55]. Ferulic acid exhibits antimicrobial, anti-allergic, hepa-protective, and antiviral effects among others [52]. Factors such as country of origin, harvest, and cultivation significantly affected only a few phenolic acids. Particularly high levels of caffeic acid were found in spring-harvested teas and those from South Korea. Sinapic acid and ellagic acid contents were significantly higher in organically grown teas and those from South Korea. 

Some researchers analysed the rich phytochemical composition of Matcha tea. They found high concentrations of chlorogenic acid (up to 4800 μg/g), synapic acid (up to 1400 μg/g), and gallic acid (up to 423 μg/g) [17]. They also found the contents of ferulic acid to be up to 289.00 μg/g of dry powder and ellagic acid to be up to 371.00 μg/g of dry powder. During water extraction, the main contributors to antioxidant activity appeared to be ellagic, chlorogenic, protocatechuic, ferulic, and p-hydroxybenzoic acids; in 80% methanol extraction, the main contributors were chlorogenic, caffeic, and p-hydroxybenzoic acids [17]. These results are consistent with the present study. However, these studies were limited, and no one has investigated the effect of various factors on the concentration of phenolic acids in powdered green tea.

It should be emphasised that different geographical conditions, climates, and storage times significantly affect its phytochemical composition and antioxidant properties. Matcha originated in Asia and is now produced in China, Japan, and South Korea. However, there have been no studies comparing the phytochemical composition of Matcha infusions from different countries. Similar results were obtained with Yerba Mate, where significant differences were noted in the chemical composition of infusions prepared from leaves of different origins [46]. 

The content of caffeine, a compound with antioxidant properties, in Matcha teas was observed by Kolackova et al. in a wide range of 14.4–34.1 mg/g [17]. In other papers, in which raw material was examined, caffeine content ranged from 5.95 to 44.43 mg/g [18]. In the study by Komes et al. the caffeine content was as high as 300.00 mg/L of aqueous extract. In our study, caffeine content ranged from 826.23 mg/L to 7313.22 mg/L. Therefore, one cup of Matcha (100 mL) can contain even 8–70 mg of caffeine. The higher values are similar to the caffeine content of black coffee. However, this was the first time more than a dozen samples of Matcha available on the food market were examined and factors related to cultivation, country of origin, and harvest time affecting the concentration of this compound were taken into account. The highest caffeine concentration was in Matcha from South Korea, organic and from the summer harvest.

Our study confirmed that country of origin influences antioxidant potential, vitamin C content, and contents of caffeine and polyphenolic compounds, especially caffeic acid, ellagic acid, sinapic acid, kaempferol, resveratrol, and rutin. Additionally, Krahe et al. confirmed differences in the concentrations of active substances in teas depending on their post-harvest storage times. Teas that had been stored longer were characterised by a lower content of antioxidant substances [29]. 

It should be emphasised that the phytochemical composition of Matcha is not fully understood and, importantly, that the impact of additional parameters, such as country of origin, cultivation method, and harvest time, was presented for the first time in our study. The hypothesis that the antioxidant potential, the total contents of polyphenols and flavonoids, and the content of vitamin C vary depending on the time of harvest, the type of cultivation, and the country of origin of Matcha tea was confirmed.

## 5. Conclusions

Matcha tea may be an important source of bioactive compounds with antioxidant activity, as confirmed by this study. Many factors influence its phytochemical composition and antioxidant properties. Type of cultivation influenced antioxidant potential, polyphenol content, vitamin C content, and ellagic acid, sinapic acid, myricetin, apigenin, resveratrol, and caffeine content. Harvest time influenced both antioxidant potential and vitamin C content as well as the contents of caffeic acid, kaempferol, quercetin, rutin, and caffeine. Country of origin influenced antioxidant potential and the contents of total polyphenols, vitamin C, caffeic acid, ellagic acid, sinapic acid, kaempferol, resveratrol, and caffeine. This study was a baseline study and provides a direction for the designing of future studies. 

## Figures and Tables

**Table 1:** Antioxidant potential (TEAC, FRAP) and phytochemical composition in different Matcha tea infusions.

Matcha	FRAP—Mm Fe(II)/L	TEAC—Trolox Equivalent (mM/L)	Polyphenols—Gallic Acid Equivalent (mg/L)	Flavonoids—Rutin Equivalent (mg/L)	Vitamin C (mg/100 mL)
**M01**	25,127.22 * ^b,c,e,h^ ± 100.11	8.87 * ^e^ ± 0.07	1083.13 * ^b,c,e,f,h^ ± 19.10	1274.18 * ^c,e,f,j,k^ ± 48.13	72.52 * ^g–k^ ± 0.57
**M02**	18,393.78 * ^a,k^ ± 272.99	7.69 * ^g,i^ ± 0.82	928.13 * ^a,d^ ± 32.51	977.42 * ^j^ ± 84.96	71.35 * ^g,i,j^ ± 0.66
**M03**	16,926.11 * ^a,d,i,k^ ± 469.77	7.12 * ^g,i,j^ ± 0.17	838.53 * ^a,d,j,k^ ± 33.74	780.92 * ^a,d,g^ ± 45.15	74.18 * ^g–k^ ± 2.90
**M04**	24,290.22 * ^c,e,h^ ± 438.20	8.28 ± 0.39	1061.66 * ^b,c,e,f,h^ ± 13.41	1359.70 * ^c,e,f,j,k^ ± 119.90	76.17 * ^g–k^ ± 1.65
**M05**	17,015.78 * ^a,d,g,k^ ± 200.75	6.39 * ^a,g,i,j,k^ ± 0.32	827.82 * ^a,d,j,k^ ± 15.61	860.77 * ^a,d^ ± 23.44	66.47 ± 3.79
**M06**	20,224.89 * ^k^ ± 1015.84	7.11 * ^g,i,j^ ± 0.57	916.36 * ^a,d^ ± 3.63	798.06 * ^a,d,g^ ± 119.40	67.82 ± 2.95
**M07**	21,692.78 * ^h^ ± 126.11	10.15 * ^b,c,e,f,h^ ± 0.12	965.08 * ^h^ ± 17.12	1217.59 * ^c,f,j,k^ ± 103.89	40.88 * ^a-d^ ± 1.85
**M08**	13,452.44 * ^a,d,g,i,k^ ± 1029.25	7.44 * ^g,i^ ± 0.15	671.29 * ^a,d,g,i,j,k^ ± 12.64	1044.63 * ^j^ ± 70.07	45.95 * ^a,c,d^ ± 5.47
**M09**	23,456.56 * ^c,e,h^ ± 1268.52	10.43 * ^b,c,e,f,h^ ± 0.30	970.18 * ^h^ ± 2.75	1024.18 * ^j^ ± 50.34	37.38 * ^a–d^ ± 3.23
**M10**	20,236.89 ± 1156.00	9.92 * ^c,e,f^ ± 0.20	1021.67 * ^c,e,h^ ± 12.34	352.36 * ^a,b,d,g,h,i^ ± 54.74	38.50 * ^a–d^ ± 1.25
**M11**	26,258.33 * ^b,c,e,f,h^ ± 1015.59	8.82 * ^e^ ± 0.45	1033.20 * ^c,e,h^ ± 6.39	787.73 * ^a,d,g^ ± 38.70	44.46 * ^a,c,d^ ± 1.88

Different letters (a–k) represent different Matcha teas: a—M01, b—M02, c—M03, d—M04, e—M05, f—M06, g—M07, h—M08, i—M-9, j—M10, and k—M11. Results are presented as mean and standard deviation values. Superscripted numbers indicate statistically significant differences in a column (*p* < 0.05). * *p* < 0.05 indicates statistically significant difference between types of Matcha tea.

**Table 2:** Antioxidant potential (TEAC, FRAP) and phytochemical composition in different Matcha tea infusions dependent on the type of cultivation (conventional or organic).

Parameter (Mean)	Cultivation	
	Organically Grown ^a^	Conventional Grown ^b^
FRAP—mM Fe(II)/L	2112.96 ± 333.51	1845.45 ± 526.77
TEAC—Trolox equivalent (mM/L)	8.26 * ^b^ ± 1.30	8.94 * ^a^ ± 1.56
Polyphenols—gallic acid equivalent (mg/L)	963.95 * ^b^ ± 90.14	820.73 * ^a^ ± 154.03
Flavonoids—rutin equivalent (mg/L)	934.30 ± 313.3	1034.40 ± 60.11
Vitamin C (mg/100 mL)	61.37 * ^b^ ± 14.93	41.66 * ^a^ ± 6.20

Different letters (a, b) represent different types of cultivation: a—organic; b—conventional. * Letters in the superscript assigned to a value represent statistically significant differences between means (*p* < 0.05). Data represent the mean and SD values.

**Table 3:** Antioxidant potential (TEAC, FRAP) and phytochemical composition in different Matcha tea infusions, dependent on the harvest time.

Parameter (Mean)	Time of Harvest		
	Spring ^c^	Summer ^d^	Unknown ^e^
FRAP—mM Fe(II)/L	2051.03 ± 309.70	1875.77 ^e *^± 481.41	21,795.41 * ^d^ ± 1646.46
TEAC—Trolox equivalent (mM/L)	7.26 * ^e^ ± 0.90	7.77 * ^e^ ± 0.80	10.17 * ^c,d^ ± 0.30
Polyphenols—gallic acid equivalent (mg/L)	935.28 ± 98.96	867.79 ^e^ ± 136.64	985.64 * ^d^ ± 134.10
Flavonoids—rutin equivalent (mg/L)	1006.18 ± 273.08	897.68 ± 132	864.71 ± 384.44
Vitamin C (mg/100 mL)	70.15 * ^e^ ± 5.25	58.99 * ^e^ ± 14.37	38.92 * ^c,d^ ± 2.64

Different letters (c, d, e) represent different times of harvest: c—spring, d—summer, and e—unknown. * Superscripted letters indicate statistically significant differences (*p* < 0.05).

**Table 4:** Antioxidant potential (TEAC, FRAP) and phytochemical composition in different Matcha tea infusions, dependent on the country of origin.

Parameter (Mean)	Country of Origin		
	China ^f^	Japan ^g^	South Korea ^h^
FRAP—mM Fe(II)/L	2266.12 * ^g^ ± 203.41	1882.49 * ^f,h^ ± 395.48	21,655.63 * ^g^ ± 3893.80
TEAC—Trolox equivalent (mM/L)	9.54 * ^h^ ± 0.98	7.65 ± 0.79	8.45 * ^f^ ± 1.62
Polyphenols—gallic acid equivalent (mg/L)	1017.83 * ^g,h^ ± 39.50	887.49 * ^f^ ± 137.43	942.03 * ^f^ ±88.12
Flavonoids—rutin equivalent (mg/L)	912.08 ± 433.91	975.04 ± 197.48	955.36 ± 201.64
Vitamin C (mg/100 mL)	50.68 * ^g^ ± 18.50	66.37 * ^f,h^ ± 10.94	50.60 * ^g^ ± 11.84

Different letters (f, g, h represent different countries of origin: f—China, g—Japan, and h—South Korea. Letters in the superscript assigned to a value represent statistically significant differences (*p* < 0.05). * *p* < 0.05 indicates statistically significant difference between countries of origin.

**Table 5:** Contents of polyphenolic acids, flavonoids, and caffeine in Matcha tea samples, divided by country of origin.

Compound	Country of Origin					
	China ^i^		Japan ^j^		South Korea ^k^	
	Median	IQR	Median	IQR	Median	IQR
4-hydroxybenzoic acid	36.32	36.04	45.96	10.06	55.58	54.47
caffeic acid	86.29	170.88	2.97 ^k^	1.05	40.69	173.83
chlorogenic acid	85.63	142.50	28.62	10.88	43.32	74.18
ellagic acid	8.82 ^k^	7.80	5.68 ^k^	6.17	14.21 ^i,j^	46.78
trans-p-coumaric acid	2.87	5.85	0.40	3.39	0.00	6.94
ferulic acid	18.48	14.82	15.22	4.79	7.61	19.41
gallic acid	478.80	856.25	247.59	241.91	62.41	954.01
sinapic acid	41.93 ^k^	50.73	28.03 ^k^	5.50	49.91 ^i,j^	59.45
apigenin	9.15	9.37	8.78	1.76	7.48	4.03
epicatechin gallate	83.79	165.96	175.03	42.06	92.10	207.93
kaempferol	4.62 ^j,k^	5.87	1.43 ^i^	0.50	1.16 ^i^	2.15
myricetin	82.44	98.71	106.15	116.81	104.32	131.03
quercetin	11.49	17.18	3.01	5.01	1.77	6.10
resveratrol	13.23 ^k^	19.11	15.31 ^k^	2.66	26.83 ^i,j^	15.47
rutin	73.94	84.10	0.00	36.64	0.00	59.82
caffeine	1397.65 ^j,k^	893.56	2125.48 ^i^	520.51	1920.10 ^i^	5379.67

Different letters (i, j, k) represent different countries of origin: i—China, j—Japan, and k—South Korea. Letters in the superscript assigned to a value represent statistically significant differences between medians (*p* < 0.05). Data represent the median and IQR values.

**Table 6:** Contents of polyphenolic acids, flavonoids, and caffeine in Matcha tea samples, divided as per the cultivation method. Data represent the median and IQR values. For apigenin and resveratrol, data are represented as means and SDs.

Compound	Type of Cultivation			
	Organic ^l^		Conventional ^m^	
	Median	IQR	Median	IQR
4-hydroxybenzoic acid	50.02	12.6	36.76	36.93
caffeic acid	3.51	103.94	3.61	3.62
chlorogenic acid	35.78	43.77	26.20	20.52
ellagic acid	11.44 ^m^	6.17	2.60 ^l^	3.88
trans-p-coumaric acid	1.57	4.66	0.20	0.40
ferulic acid	16.39	16.33	12.06	1.71
gallic acid	152.62	550.05	180.66	247.80
sinapic acid	32.50 ^m^	30.59	11.62 ^l^	7.94
epicatechin gallate	171.93	161.33	80.78	171.87
kaempferol	1.54	2.90	1.31	0.57
myricetin	114.30 ^m^	84.11	15.28 ^l^	31.21
quercetin	3.43	5.65	2.98	0.19
rutin	16.99	48.60	14.12	31.06
caffeine	2065.57 ^m^	622.27	1395.91 ^l^	893.59
	**Mean**	**SD**	**Mean**	**SD**
apigenin	9.23 ^m^	2.31	6.62 ^l^	0.41
resveratrol	21.66 ^m^	7.00	9.20 ^l^	6.02

Different letters (l, m) represent different types of cultivation: l—organic; m—conventional. Letters in the superscript assigned to a value represent statistically significant differences between medians (*p* < 0.05). For apigenin and resveratrol, letters in the superscript assigned to a value represent statistically significant differences between means (*p* < 0.05). Data represent the median and IQR values.

**Table 7:** Contents of polyphenolic acids, flavonoids, and caffeine in Matcha tea samples, divided by time of harvest.

Compound	Time of Harvest					
	Spring ^n^		Summer ^o^		Unknown ^p^	
	Median	IQR	Median	IQR	Median	IQR
4-hydroxybenzoic acid	52.93	15.07	44.19	4.01	53.75	68.19
caffeic acid	3.93 ^o^	2.51	2.14 ^n,p^	0.87	40.69 ^o^	169.23
chlorogenic acid	40.69	17.31	36.95	1.11	27.60	138.14
ellagic acid	5.68	2.78	11.44	1.04	12.55	49.42
trans-p-coumaric acid	0.00	0.41	3.45	0.31	0.00	5.76
ferulic acid	12.36	15.07	16.39	1.49	10.65	17.46
gallic acid	37.99	277.07	266.61	24.16	62.61	842.69
sinapic acid	28.22	10.65	30.80	2.79	66.93	77.62
apigenin	7.48	4.14	8.77	0.80	10.67	8.76
epicatechin gallate	191.22	206.40	174.84	15.85	92.10	153.92
kaempferol	1.10 ^p^	0.22	1.55	0.14	3.00 ^n^	5.60
myricetin	104.32	144.39	114.30	10.36	32.34	123.49
quercetin	0.47 ^o,p^	3.06	5.58 ^n^	0.51	2.91 ^n^	18.02
resveratrol	17.43	10.22	15.30	1.39	22.78	29.50
rutin	0.00 ^o,p^	0.00	37.34 ^n^	3.38	31.06 ^n^	113.43
caffeine	1802.66 ^o^	615.40	2336.11 ^n,p^	212.60	1802.62 ^o^	5707.06

Different letters (n, o, p) represent times of harvest: n—Spring, o—Summer, and p—Unknown. Letters in the superscript assigned to a value represent statistically significant differences between medians (*p* < 0.05). Data represent the median and IQR values.

## Data Availability

The original contributions presented in the study are included in the article/Supplementary Material, further inquiries can be directed to the corresponding author.

## Supplementary Materials

The following supporting information can be downloaded at: https://www.mdpi.com/article/10.3390/foods13081270/s1, Table S1: Characteristics of examined material available on the Polish food market, Table S2: Spearman’s rank correlations between parameters for Matcha tea, Table S3: Summary of the contents of polyphenolic acids, flavonoids, and caffeine in Matcha tea samples. Data represent the minimum, 25th percentile, median, 75th percentile, and maximum values.
