# Supplementary material for: Exploring the Influence of Origin, Harvest Time, and Cultivation Method on Antioxidant Capacity and Bioactive Compounds of Matcha Teas
Source: Foods. 2024 Apr 21;13(8):1270. doi: 10.3390/foods13081270 (PMC11048880; doi:10.3390/foods13081270)
Supplement: Supplementary file 1 [file foods-13-01270-s001.zip › foods-2960694-supplementary.pdf]

**Table S1.** Characteristics of  examined material. available on the Polish food market.

| Material | Cultivation | Time of harvest | Country of origin | Supplier |
|---|---|---|---|---|
| M1 | organically grown | summer | Japan | Moya Matcha |
| M2 | organically grown | summer | Japan | Moya Matcha |
| M3 | organically grown | spring | Japan | Moya Matcha |
| M4 | organically grown | spring | China | Oxalis |
| M5 | organically grown | spring | South Korea | Oxalis |
| M6 | organically grown | spring | Japan | Oxalis |
| M7 | organically grown | unknown | South Korea | Brown House & Tea |
| M8 | conventional | spring | Japan | Ju Matcha |
| M9 | conventional | unknown | China | Czas na herbatę |
| M10 | organically grown | unknown | China | Superfoods |
| M11 | organically grown | spring | South Korea | Nokchawon |

**Table S2.** Spearman's rank correlation between parameters for Matcha tea.

| | | 4-hydroxybenzoic acid | apigenin | caffeic acid | caffeine | chlorogenic acid | ellagic acid | epicatechin gallate | ferulic acid | flavonoids | FRAP | gallic acid | kaempferol | myricetin | p-coumaric acid | polyphenols | quercetin | resveratrol | rutin | sinapic acid | TEAC | vitamin C |
|---|---|---|---|---|---|---|---|---|---|---|---|---|---|---|---|---|---|---|---|---|---|---|
| 4-hydroxybenzoic acid | Correlation coefficient | 1 | - | - | - | - | - | - | - | - | - | - | - | - | - | - | - | - | - | - | - | - |
| | Significance Level P | - | - | - | - | - | - | - | - | - | - | - | - | - | - | - | - | - | - | - | - | - |
| apigenin | Correlation coefficient | 0.408 | 1 | - | - | - | - | - | - | - | - | - | - | - | - | - | - | - | - | - | - | - |
| | Significance Level P | 0.0251 | - | - | - | - | - | - | - | - | - | - | - | - | - | - | - | - | - | - | - | - |
| caffeic acid | Correlation coefficient | 0.64 | 0.179 | 1 | - | - | - | - | - | - | - | - | - | - | - | - | - | - | - | - | - | - |
| | Significance Level P | 0.0001 | 0.3437 | - | - | - | - | - | - | - | - | - | - | - | - | - | - | - | - | - | - | - |
| caffeine | Correlation coefficient | 0.642 | 0.481 | -0.001 | 1 | - | - | - | - | - | - | - | - | - | - | - | - | - | - | - | - | - |
| | Significance Level P | 0.0001 | 0.0071 | 0.9953 | - | - | - | - | - | - | - | - | - | - | - | - | - | - | - | - | - | - |
| chlorogenic acid | Correlation coefficient | **0.847** | 0.592 | 0.651 | 0.56 | 1 | - | - | - | - | - | - | - | - | - | - | - | - | - | - | - | - |
| | Significance Level P | <0.0001 | 0.0006 | 0.0001 | 0.0013 | - | - | - | - | - | - | - | - | - | - | - | - | - | - | - | - | - |
| ellagic acid | Correlation coefficient | 0.162 | 0.545 | 0.235 | 0.382 | 0.255 | 1 | - | - | - | - | - | - | - | - | - | - | - | - | - | - | - |
| | Significance Level P | 0.3919 | 0.0019 | 0.211 | 0.037 | 0.1743 | - | - | - | - | - | - | - | - | - | - | - | - | - | - | - | - |
| epicatechin gallate | Correlation coefficient | 0.063 | 0.103 | -0.2 | 0.225 | -0.009 | -0.197 | 1 | - | - | - | - | - | - | - | - | - | - | - | - | - | - |
| | Significance Level P | 0.741 | 0.5897 | 0.2882 | 0.2312 | 0.9618 | 0.297 | - | - | - | - | - | - | - | - | - | - | - | - | - | - | - |
| ferulic acid | Correlation coefficient | -0.273 | 0.277 | -0.233 | -0.111 | -0.047 | 0.254 | -0.346 | 1 | - | - | - | - | - | - | - | - | - | - | - | - | - |
| | Significance Level P | 0.1451 | 0.1377 | 0.2146 | 0.5592 | 0.8054 | 0.1759 | 0.0608 | - | - | - | - | - | - | - | - | - | - | - | - | - | - |
| flavonoids | Correlation coefficient | 0.106 | -0.229 | -0.341 | 0.561 | -0.112 | -0.007 | 0.034 | -0.471 | 1 | - | - | - | - | - | - | - | - | - | - | - | - |
| | Significance Level P | 0.5784 | 0.2237 | 0.0651 | 0.0013 | 0.5545 | 0.9693 | 0.8601 | 0.0087 | - | - | - | - | - | - | - | - | - | - | - | - | - |
| FRAP | Correlation coefficient | -0.549 | 0.028 | -0.228 | -0.18 | -0.425 | 0.533 | -0.231 | 0.18 | 0.295 | 1 | - | - | - | - | - | - | - | - | - | - | - |
| | Significance Level P | 0.0017 | 0.884 | 0.2265 | 0.3413 | 0.0193 | 0.0024 | 0.2191 | 0.3425 | 0.003 | - | - | - | - | - | - | - | - | - | - | - | - |
| gallic acid | Correlation coefficient | -0.124 | -0.067 | 0.081 | -0.106 | -0.037 | 0.415 | -0.749 | 0.648 | -0.102 | 0.29 | 1 | - | - | - | - | - | - | - | - | - | - |
| | Significance Level P | 0.5141 | 0.7252 | 0.6697 | 0.5768 | 0.8473 | 0.0225 | <0.0001 | 0.0001 | 0.5929 | 0.1196 | - | - | - | - | - | - | - | - | - | - | - |
| kaempferol | Correlation coefficient | 0.371 | 0.633 | -0.024 | 0.447 | 0.574 | 0.212 | 0.22 | 0.174 | -0.031 | -0.032 | -0.097 | 1 | - | - | - | - | - | - | - | - | - |
| | Significance Level P | 0.0434 | 0.0002 | 0.9006 | 0.0133 | 0.0009 | 0.2617 | 0.2427 | 0.3585 | 0.8693 | 0.8656 | 0.6093 | - | - | - | - | - | - | - | - | - | - |
| myricetin | Correlation coefficient | 0.152 | 0.308 | 0.058 | 0.269 | 0.347 | 0.211 | 0.551 | 0.363 | -0.357 | -0.119 | -0.054 | 0.462 | 1 | - | - | - | - | - | - | - | - |
| | Significance Level P | 0.4242 | 0.0981 | 0.7623 | 0.15 | 0.0601 | 0.2638 | 0.0016 | 0.0485 | 0.0531 | 0.5295 | 0.7766 | 0.0102 | - | - | - | - | - | - | - | - | - |
| p-coumaric acid | Correlation coefficient | -0.269 | 0.108 | -0.05 | -0.075 | -0.09 | 0.461 | -0.578 | **0.781** | -0.209 | 0.326 | **0.897** | -0.131 | 0.116 | 1 | - | - | - | - | - | - | - |
| | Significance Level P | 0.1507 | 0.571 | 0.7923 | 0.6941 | 0.6376 | 0.0104 | 0.0008 | <0.0001 | 0.2666 | 0.0783 | <0.0001 | 0.4886 | 0.5423 | - | - | - | - | - | - | - | - |
| polyphenols | Correlation coefficient | -0.535 | 0.198 | -0.201 | -0.18 | -0.288 | 0.541 | -0.3 | 0.394 | 0.299 | **0.869** | 0.374 | 0.09 | -0.009 | 0.476 | 1 | - | - | - | - | - | - |
| | Significance Level P | 0.0023 | 0.294 | 0.288 | 0.3404 | 0.1227 | 0.002 | 0.1075 | 0.0312 | 0.0026 | <0.0001 | 0.0415 | 0.6374 | 0.9637 | 0.0079 | - | - | - | - | - | - | - |
| quercetin | Correlation coefficient | -0.257 | 0.102 | -0.129 | -0.045 | -0.047 | 0.465 | -0.59 | **0.763** | -0.113 | 0.418 | **0.924** | 0.153 | 0.133 | **0.91** | 0.551 | 1 | - | - | - | - | - |
| | Significance Level P | 0.171 | 0.5911 | 0.4958 | 0.8144 | 0.8072 | 0.0097 | 0.0006 | <0.0001 | 0.5511 | 0.0215 | <0.0001 | 0.4185 | 0.4839 | <0.0001 | 0.0016 | - | - | - | - | - | - |
| resveratrol | Correlation coefficient | **0.752** | 0.584 | 0.628 | 0.498 | **0.707** | 0.523 | 0.254 | -0.095 | -0.234 | -0.234 | -0.175 | 0.354 | 0.454 | -0.186 | -0.228 | -0.229 | 1 | - | - | - | - |
| | Significance Level P | <0.0001 | 0.0007 | 0.0002 | 0.0051 | <0.0001 | 0.0031 | 0.1751 | 0.6175 | 0.2128 | 0.2137 | 0.356 | 0.0553 | 0.0117 | 0.3246 | 0.2262 | 0.2244 | - | - | - | - | - |
| rutin | Correlation coefficient | -0.475 | 0.156 | -0.205 | -0.202 | -0.19 | 0.493 | -0.352 | **0.728** | -0.271 | 0.554 | **0.747** | 0.146 | 0.263 | **0.85** | 0.686 | **0.91** | -0.247 | 1 | - | - | - |
| | Significance Level P | 0.008 | 0.4112 | 0.2767 | 0.2836 | 0.3134 | 0.0056 | 0.0567 | <0.0001 | 0.1477 | 0.0015 | <0.0001 | 0.4407 | 0.1607 | <0.0001 | <0.0001 | <0.0001 | 0.189 | - | - | - | - |
| sinapic acid | Correlation coefficient | 0.341 | 0.585 | 0.485 | 0.353 | 0.442 | **0.943** | -0.087 | 0.114 | -0.151 | 0.361 | 0.285 | 0.262 | 0.308 | 0.316 | 0.376 | 0.305 | **0.703** | 0.362 | 1 | - | - |
| | Significance Level P | 0.0651 | 0.0007 | 0.0066 | 0.056 | 0.0146 | <0.0001 | 0.6493 | 0.5497 | 0.4256 | 0.05 | 0.1263 | 0.1622 | 0.0976 | 0.0889 | 0.0404 | 0.1016 | <0.0001 | 0.0496 | - | - | - |
| TEAC | Correlation coefficient | -0.078 | 0.101 | -0.064 | 0.082 | 0.044 | 0.501 | -0.468 | 0.16 | 0.259 | 0.61 | 0.463 | 0.436 | -0.146 | 0.237 | 0.566 | 0.552 | -0.051 | 0.461 | 0.378 | 1 | - |
| | Significance Level P | 0.6817 | 0.597 | 0.7383 | 0.6671 | 0.8154 | 0.0048 | 0.0091 | 0.3978 | 0.0096 | <0.0001 | 0.0099 | 0.016 | 0.4429 | 0.2067 | <0.0001 | 0.0015 | 0.7901 | 0.0104 | 0.0396 | - | - |
| vitamin C | Correlation coefficient | 0.077 | -0.063 | -0.33 | 0.56 | -0.057 | -0.143 | 0.529 | -0.21 | 0.24 | -0.036 | -0.354 | -0.178 | 0.243 | -0.133 | 0.058 | -0.306 | -0.025 | -0.299 | -0.201 | -0.535 | 1 |
| | Significance Level P | 0.6877 | 0.7401 | 0.0752 | 0.0013 | 0.765 | 0.4514 | 0.0026 | 0.2663 | 0.0169 | 0.724 | 0.0548 | 0.3479 | 0.1953 | 0.483 | 0.571 | 0.1002 | 0.8969 | 0.1089 | 0.2865 | <0.0001 | - |

**Table S3.** Summary of the content of polyphenolic acids. flavonoids and caffeine in Matcha tea samples. Data represent the minimum. 25th percentile. median. 75th percentile and maximum values.

| **Compound** | **Minimum [mg/L]** | **25th percentile [mg/L]** | **Median [mg/L]** | **75th percentile [mg/L]** | **Maximum [mg/L]** | **IQR** |
|---|---|---|---|---|---|---|
| **4-hydroxybenzoic acid** | 16.10 | 40.22 | 50.02 | 54.85 | 95.56 | 14.63 |
| **caffeic acid** | 1.65 | 2.60 | 3.51 | 9.94 | 185.94 | 7.34 |
| **chlorogenic acid** | 15.61 | 26.69 | 32.54 | 43.81 | 164.08 | 17.12 |
| **ellagic acid** | 0.85 | 5.25 | 9.17 | 12.55 | 59.58 | 7.30 |
| **trans-p-coumaric acid** | 0.00 | 0.00 | 0.20 | 3.52 | 7.82 | 3.52 |
| **ferulic acid** | 6.60 | 8.47 | 13.83 | 22.46 | 29.63 | 13.99 |
| **gallic acid** | 13.85 | 37.99 | 153.01 | 304.02 | 1119.42 | 266.03 |
| **sinapic acid** | 8.09 | 26.09 | 30.80 | 49.91 | 104.03 | 23.82 |
| **apigenin** | 3.93 | 5.57 | 8.72 | 9.94 | 13.97 | 4.37 |
| **epicatechin gallate** | 2.68 | 8.66 | 165.79 | 196.84 | 234.03 | 188.18 |
| **kaempferol** | 0.54 | 1.10 | 1.52 | 3.00 | 7.68 | 1.9 |
| **myricetin** | 1.11 | 5.58 | 105.23 | 131.05 | 153.33 | 125.47 |
| **quercetin** | 0 | 0.47 | 2.98 | 5.69 | 20.50 | 5.22 |
| **resveratrol** | 3.33 | 14.63 | 16.71 | 25.32 | 36.57 | 10.69 |
| **rutin** | 0.00 | 0.00 | 14.12 | 38.06 | 117.51 | 38.06 |
| **caffeine** | 826.23 | 1522.57 | 1853.20 | 2182.88 | 7313.22 | 660.31 |
